# Genomic signatures of domestication on neurogenetic genes in *Drosophila melanogaster*

**DOI:** 10.1186/s12862-015-0580-1

**Published:** 2016-01-05

**Authors:** Craig E. Stanley, Rob J. Kulathinal

**Affiliations:** Department of Biology, Temple University, Philadelphia, PA USA

**Keywords:** Behavior, Adaptation, Purifying selection, Positive selection, Extended haplotypes, Domestication genomics, Model organisms, Domestication syndrome

## Abstract

**Background:**

Domesticated animals quickly evolve docile and submissive behaviors after isolation from their wild conspecifics. Model organisms reared for prolonged periods in the laboratory also exhibit similar shifts towards these domesticated behaviors. Yet whether this divergence is due to inadvertent selection in the lab or the fixation of deleterious mutations remains unknown.

**Results:**

Here, we compare the genomes of lab-reared and wild-caught *Drosophila melanogaster* to understand the genetic basis of these recently endowed behaviors common to laboratory models. From reassembled genomes of common lab strains, we identify unique, derived variants not present in global populations (lab-specific SNPs). Decreased selective constraints across low frequency SNPs (unique to one or two lab strains) are different from patterns found in the wild and more similar to neutral expectations, suggesting an overall accumulation of deleterious mutations. However, high-frequency lab SNPs found in most or all lab strains reveal an enrichment of X-linked loci and neuro-sensory genes across large extended haplotypes. Among shared polymorphisms, we also find highly differentiated SNPs, in which the derived allele is higher in frequency in the wild (Fst*_wild>lab_), enriched for similar neurogenetic ontologies, indicative of relaxed selection on more active wild alleles in the lab.

**Conclusions:**

Among random mutations that continuously accumulate in the laboratory, we detect common adaptive signatures in domesticated lab strains of fruit flies. Our results demonstrate that lab animals can quickly evolve domesticated behaviors via unconscious selection by humans early on a broad pool of disproportionately large neurogenetic targets followed by the fixation of accumulated deleterious mutations on functionally similar targets.

**Electronic supplementary material:**

The online version of this article (doi:10.1186/s12862-015-0580-1) contains supplementary material, which is available to authorized users.

## Background

Our recent history of domesticating plants and animals [1] offers a diversity of genetic systems to study evolution in action [2]. Crop and livestock breeders often provoke relatively large phenotypic changes across successive generations conditioned on available standing genetic variation found in wild progenitor populations. Such changes demonstrate the formidable power of directional selection over relatively short periods of time. In fact, Charles Darwin devoted the opening chapter of “On the origins of species” to artificial selection in order to introduce natural selection as the principal driver of evolutionary change [3]. In his two-volume follow-up devoted specifically to domestication, Darwin noted that “selection may be followed either methodically and intentionally, or unconsciously and unintentionally” [4]. These histories can also be modeled as a co-evolutionary framework between humans and the crops and livestock they cultivate [5], whether the selective pressures were intentional or not.

While domesticates usually have reduced effective population sizes relative to their ancestral populations, there still remains ample variation for selection to act upon. Numerous loci involved in animal and crop domestication are found to harbor positive selection coefficients [6] and selectively swept regions [7]. In domestic chickens, genomic sequences from multiple lines reveal the presence of selective sweeps leading to the discovery of causative agents in growth differences between domestic lines [8]. In household pets, selection for certain behavioral and sensory traits [9] have produced signatures of positive selection in vision and hearing genes in domesticated cats [10] and neural development genes in dogs [11]. The growing literature on crop, livestock, and pet domestication reveals that long-term selection by humans can generate strong signals of selection at the genomic level and provides a new lens into the strength and target of selection during our recent domesticated past.

**Fig. 1:**
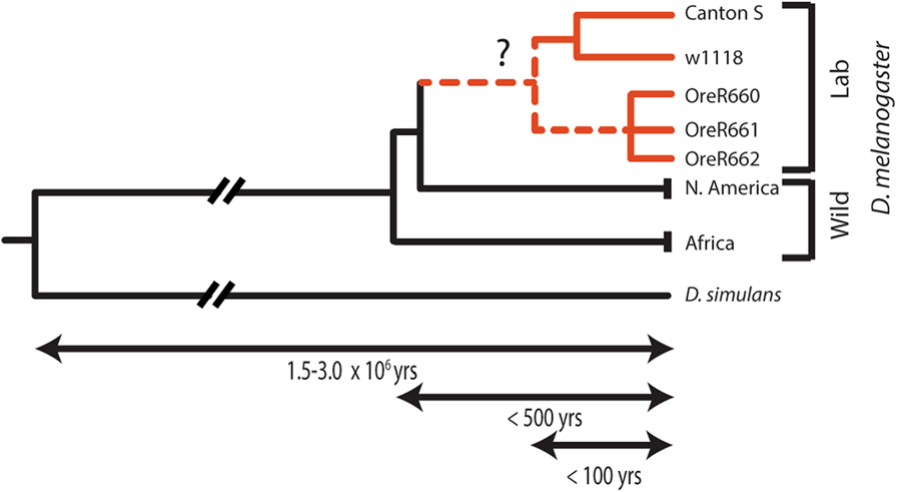
Hypothetical genealogical relationship of laboratory strains in relation to global populations of *D. melanogaster* and the closely related species, *D. simulans*. The five lab strains are indicated in red with hypothetical ancestral relationships marked as a dotted line. Estimated time since common ancestors are indicated below

Animals bred in the laboratory as model organisms may also be adapted to human conditions, and over a much shorter time period. Studies comparing laboratory strains of mice and nematodes have identified genetic differences in genes involved in behavior [12] and metabolism [13, 14] suggesting adaptation to novel conditions in the laboratory (e.g., [15]). Over a century ago, *Drosophila melanogaster* was brought into the laboratory initially as a teaching tool [16], and its fast generation time and relative ease of maintenance quickly made the fruit fly an important genetics research tool [17] in such varied fields such as development, physiology, and evolution. Canton-S(pecial), the oldest known wildtype fly stock, was captured by Calvin Bridges over a century ago from a natural population in Canton, Ohio, and first debuted in his seminal 1916 paper on “Non-disjunction as proof of the chromosome theory of heredity” [18]. Approximately a decade later, Donald Lancefield, another product of Thomas Morgan’s prolific lab at Columbia University, extracted Oregon-R from a population in Roseburg, Oregon [19]. Many commonly used fly stocks eventually coalesce ancestrally to these old North American laboratory stalwarts, Canton-S and Oregon-R (Fig. 1), which were independently extracted from populations in North America and themselves possessing relatively recent African origins ([20]; Fig. 1).

With at least a dozen generations per year, *D. melanogaster* lab stocks have been isolated from their wild ancestors for over a thousand generations, providing ample time to sufficiently diverge. It is known among fruit fly researchers that behavioral traits of laboratory *vs*. wild *D. melanogaster* are distinct, with lab-reared flies far tamer and more manageable than those found in the wild. Although lab-reared flies are generally maintained under relatively standard conditions of temperature, light, and diet, selective pressures in the lab are very different than those found in the wild providing the potential for strong selection for human-accommodating phenotypes and/or the relaxation of selection on traits adapted in nature [21, 22]. On the other hand, domesticates are generally less reproductively fit than their wild relatives [23] and may exhibit similar less optimal behavioral phenotypes. Lab stocks typically experience drastic decreases in population size and higher levels of inbreeding ultimately decreasing the efficacy of selection to rid lines of continually re-occurring deleterious alleles [24]. Whether these phenotypic differences in lab strains are driven by conscious selection, inadvertent selection, a relaxation in selection, or are simply the fixation of deleterious mutations, is not known.

Its relatively recent and well-documented history, access to multiple isolated lines reared in similar environmental settings, well-characterized functional annotations, and the availability of hundreds of genomes from extensively sampled extant populations including a closely related species, make *D. melanogaster* an ideal model system to study the evolutionary processes that underlie rapid phenotypic change. Here, we investigate genetic differences between common laboratory stains of *D. melanogaster* to those recently caught from nature to examine whether this important, centuries-old genetic model has evolved convergent domesticated behaviors by adapting to a bottled existence or being inundated by low-fitness alleles. We first document behavioral differences between lab-reared and wild-caught flies with respect to their general activity and locomotory abilities. We then identify SNPs that are differentiated between the genomes of laboratory and wild strains to evaluate the roles of selection *vs*. drift in flies isolated in bottles. Among uniquely derived alleles found only in lab strains we find patterns of drift and selection across, respectively, low and high frequency classes, when comparing evolutionary parameters such as Grantham distance, missense to silent mutations, positional distribution within codons, and levels of codon bias. We further characterize putative regions under sustained selection among lab strains and find longer than expected haplotypes near high-frequency derived SNPs, also enriched in neuro-sensory genes. Finally, we suggest that this neurogenetic class, residing on a disproportionately large fraction of the genome relative to other functional classes, provides a large mutational target for genetic variation to accumulate and selection to act upon. Thus, the genomics of fly domestication reveal an interplay of evolutionary forces with mutation and selection on a large neurogenetic class of genes playing a pivotal role in *D. melanogaster*’s brief, but distinguished, history in the laboratory.

## Results

### Behavior

Although it is well-known among Drosophilists that lab strains are much slower and easier to handle than live flies, literature documenting these differences is lacking. We first confirm anecdotal reports of differences in the overall activity between laboratory stocks (Canton-S, Oregon-R, w^1118^) and flies from wild populations (Additional file 1: Figure S1). On average, flies from lab strains are significantly less active than wild-caught individuals using different measures of locomotion including standard and angular velocities (Additional file 1: Figure S1A,B; Wilcoxon *P < 0.05*) and time-spent moving *vs*. stationary (Additional file 1: Figures S1C, S1D; Wilcoxon *P < 0.05)*. Lab strains also demonstrate less responsiveness in the form of interactions between individuals compared to wild-caught lines where the latter’s movement is more uniformly distributed and gradually increases with proximity to neighbors (Additional file 1: Figure S1E). Flies from laboratory strains do not appear to follow this relationship with motion uncorrelated to the proximity with their nearest neighbor (Additional file 1: Figure S1F). Overall, these results provide general support for a convergence of slow-moving and less responsive behavior in lab strains.

### Genetic variation in lab strains

**Fig. 2:**
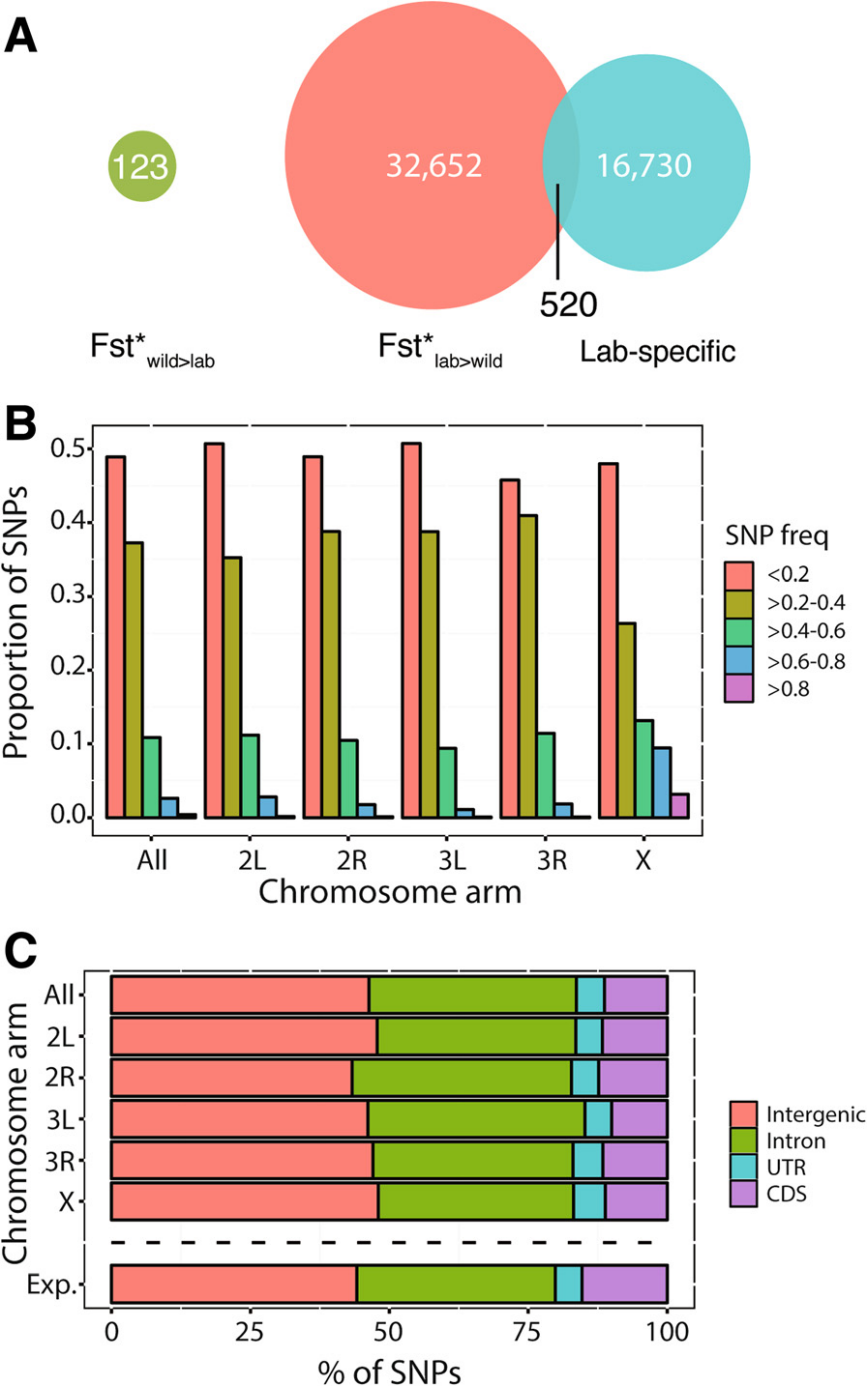
Characterization of derived SNPs differentiated between lab strains and a wild *D. melanogaster* population. **a** Number and overlap of lab-specific SNPs (derived and unique among lab strains) with Fst*_lab>wild_ SNPs (derived allele is higher in frequency among lab strains compared to sequenced flies from Raleigh NC). The total number of Fst*_wild>lab_ SNPs (the derived allele is shared and lower in frequency among lab strains) is also shown. **b** Site frequency distribution of lab-specific SNPs, per chromosome. **c** Genic characterization of lab-specific SNPs found in the majority (at least 3/5) of laboratory strains, across chromosomal arms

After applying filters for quality and missing data, a total of 98,442,787 base pairs were analyzed across five reassembled laboratory strains of *D. melanogaster*, 516 genomes from 23 global populations including an extensively sampled population from North America (*n* = 205), and one closely related species, *D. simulans*. Among 14,545,645 polymorphic sites, 68.5 % are non-singletons and used for subsequent analyses (Additional file 2: Table S1). To test for signals of domestication, we used three defined categories of SNPs differentiated between lab strains and populations of *D. melanogaster* from nature: lab-specific SNPs and two types of highly differentiated F_ST_ (Fst*) SNPs, depending on whether the derived allele is found at a higher frequency among lab strains (Fst*_lab>wild_), or in a representative natural population (Fst*_wild>lab_). While distinct, some overlap exists between the lab-specific and Fst*_lab>wild_ analysis categories: out of a total 50,565 differentiated SNPs, 520 SNPs are common to these two SNP classes (Fig. 2a). In contrast, Fst*_wild>lab_ SNPs are unique and relatively rare (*n* = 123; Fig. 2a).

#### Lab-specific SNPs

The number of lab-specific SNPs is 17,250, with 9,258 and 1,951 SNPs located in genic and CDS regions, respectively (Fig. 2c). The total number of lab-specific SNPs is significantly larger than a subsample of five random genomes from North Carolina and comparison of the number of SNPs specific to this random subset against the entire North Carolinian dataset (performed 1,000 times; Sign test, *P < 0.002*) as well as the entire global dataset (Sign test, *P < 0.001*). Lab-specific SNPs represent only a small fraction (0.017 %) of the total SNP variation found among global populations of *D. melanogaster*, including singletons. Lab-specific SNPs are distributed across all chromosome arms with a slight but significant enrichment across non-coding regions (e.g., intergenic, 1.02x, χ^2^ = 33.56, *P < 0.001*; intronic, 1.05x, χ^2^ = 19.11, *P < 0.001;* Fig. 2b, c). The X-chromosome is enriched for mid- and high-frequency lab-specific SNPs (χ^2^ = 201.48, *P < 0.001*). The number of fixed lab-specific SNPs (i.e., found in all five lab strains, or 4/4 due to missing data) was 75, 46, and 32, respectively, in all genomic regions, genic, and CDS regions (Additional file 2: Table S1). Genes containing fixed lab-specific SNPs are enriched for regulatory and signaling gene ontology (GO) categories (Additional file 3: Table S2; Additional file 4: Table S3) and up-regulated in neural-sensory tissues (Additional file 5: Figure S2).

#### Fst*_lab>wild_ SNPs

The number of derived SNPs with high F_ST_ (i.e., Fst*) that harbor a greater frequency in the lab compared to the wild is 33,172, with 18,284 and 4,116 in genic and CDS regions respectively (Additional file 2: Table S1). Fst*_lab>wild_ SNPs generally describe variants that are highly represented (≥80 %) among lab strains but have a low (<0.35) allele frequency in global populations. Fst*_lab>wild_ are uniformly distributed across chromosome arms and are enriched for genic regions (regulatory, 1.07x, χ^2^ = 104.09, *P < 0.001*; Additional file 2: Table S1). The number of Fst*_lab>wild_ SNPs that are fixed (i.e., found in all lab strains) is 30,396 and 3,829, respectively, in all genomic regions and CDS regions (Additional file 2: Table S1). Fst*_lab>wild_ SNPs are functionally enriched in general developmental gene ontology categories (100+ GO categories are significantly enriched; Additional file 3: Table S2; Additional file 4: Table S3). Fst*_lab>wild_ SNPs are upregulated in several tissues including ganglion, larval CNS, and ovary (Additional file 5: Figure S2).

#### Fst*_wild>lab_ SNPs

A small proportion of Fst* SNPs (*n* = 123) are highly differentiated in the opposite direction. These Fst*_wild>lab_ SNPs are found at low frequency (≤25 %) among lab strains but have high allele frequencies in global populations (>93 %). Fst*_wild>lab_ SNPs are enriched on the X-chromosome (1.6x, χ^2^ = 9.45, *P < 0.01*), across intergenic regions (1.25x, χ^2^ = 4.14, *P < 0.05*), and significantly enriched for neurogenetic genes (3.6x, χ^2^ = 28.73, *P < 0.001*; Additional file 3: Table S2). Specifically, this SNP category is significantly enriched in nervous system development and photoreceptor development gene ontologies and is upregulated in neural, visual, and ovarian tissues (Additional file 4: Table S3, Additional file 5: Figure S2).

### Evolutionary relationships

Phylogenetic analyses on lab-specific SNPs and Fst* SNPs were performed to provide insight on the origin and relationship among isolated lab stocks (Additional file 6: Figure S3). The lab-specific consensus SNP tree reveals Canton-S as ancestral to the three Oregon-R strains and the w^1118^ strain, as expected (Additional file 6: Figure S3A). The Fst* SNP consensus tree similarly shows a distinct lab monophyletic clade with similar bootstrap support for lab strain topology (Additional file 6: Figure S3B). A distinct origin among lab strains is also seen using a random set of 100,000 polymorphic sites among lab and wild strains (Additional file 6: Figure S3C). The branch lengths of the lab strains dramatically differ between trees indicating each lab strain’s distinctiveness (with the exception of OreR-661 and OreR-662) from each other (Additional file 6: Figure S3A) and the extant North American population (Additional file 6: Figure S3B). The three congruent phylogenetic trees also revealed several surprises including the three Oregon-R strains being paraphyletic, with Ore-661 and Ore-662 more similar to w^1118^ than Ore-660. Also, while the original Oregon-R strain was independently sampled on the US west coast a few decades later, all lab strains appear to be derived from a single Canton-S common ancestor (Additional file 6: Figure S3C), with the lab clade best supported next to extant populations from France, then North America.

### Genome-wide levels of selection

**Fig. 3:**
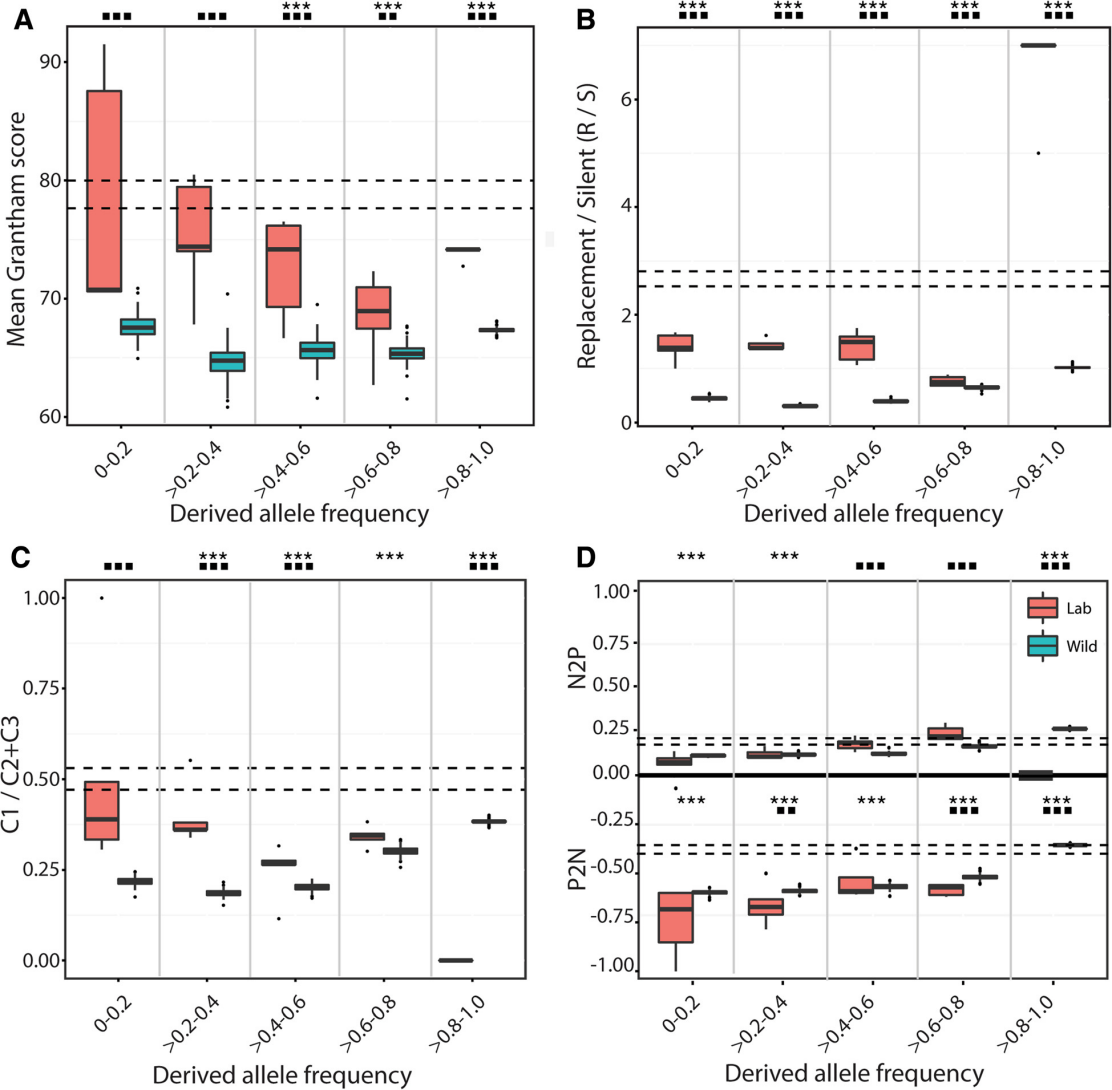
Frequency distribution of lab-specific (red), wild-caught (green), and neutrally simulated derived (between dotted lines) SNPs across various evolutionary parameters. **a** Mean Grantham score per replacement substitution. **b** Mean replacement/silent (R/S) ratio per SNP per individual. **c** Mean ratio of the number of first codon position (C1) SNPs to the number of second and third codon position (C2+C3) SNPs. **d** Mean proportion of non-preferred to preferred (N2P) codons and preferred to non-preferred (P2N) codons per individual. For all panels, dotted black horizontal lines represent one standard deviation from the mean of 1000 random simulations using *D. simulans* sequence data (matched to the number of lab-specific SNPs). Statistical significance determined using a Wilcoxon rank-sum test. Asterisks indicate significant differences between lab-specific SNPs and SNPs derived from neutral simulations (***P-value < 0.001, ** *P*-value < 0.05). Squares indicate significant differences between lab-specific SNPs and SNPs derived from the Raleigh NC population (■■■*P*-value < 0.001, ■■*P*-value < 0.05)

To detect differences in selective constraints in the lab, we estimate a series of evolutionary parameters (Grantham distance, R/S ratio, C1/C2+C3, codon bias) across five frequency bins of lab-specific SNPs located in coding regions. We compare parameter estimates against similarly binned derived SNPs found only in the Raleigh NC population along with simulation estimates expected under strict neutrality. Additional file 2: Table S1 compares the nature and number of lab-differentiated SNPs to those found in the Raleigh NC population across autosomes and X-chromosomes. When not binned according to allele frequency, mean Grantham scores, R/S, and C1/(C2+C3) ratios are similar to random simulations. However, when grouped by frequency class, parameter estimates of medium to high frequency SNPs more closely follow a pattern of similarly binned SNPs from the wild rather than expected neutral patterns based on a random mutational model (Fig. 3).

### Effective population size

The effective population size is a strong determinant of the effectiveness of selection and drift [25, 26]. Watterson’s theta, Θ_s_ [27], was estimated across non-overlapping 50 kb windows to compare the amount of genetic variation from an extant population to that found among all lab strains. Like previous genome-wide estimates of nucleotide diversity from the Raleigh NC population [28–30], our estimates of Θ similarly fluctuate across genomic regions with a genome-wide average of Θ_NC_ = 0.0053 (Additional file 7: Figure S4). Although laboratory strains do not collectively comprise a true interbreeding population, we estimate Θ_lab_ to provide a relative measure of ancestral N_e_. Laboratory nucleotide diversity varies along the genome in a similar fashion as the Raleigh NC population with a genome-wide mean of Θ_lab_ = 0.00312 (Additional file 7: Figure S4), indicating at least a two-fold reduction in ancestral N_e_.

### Extended haplotype blocks

**Fig. 4:**
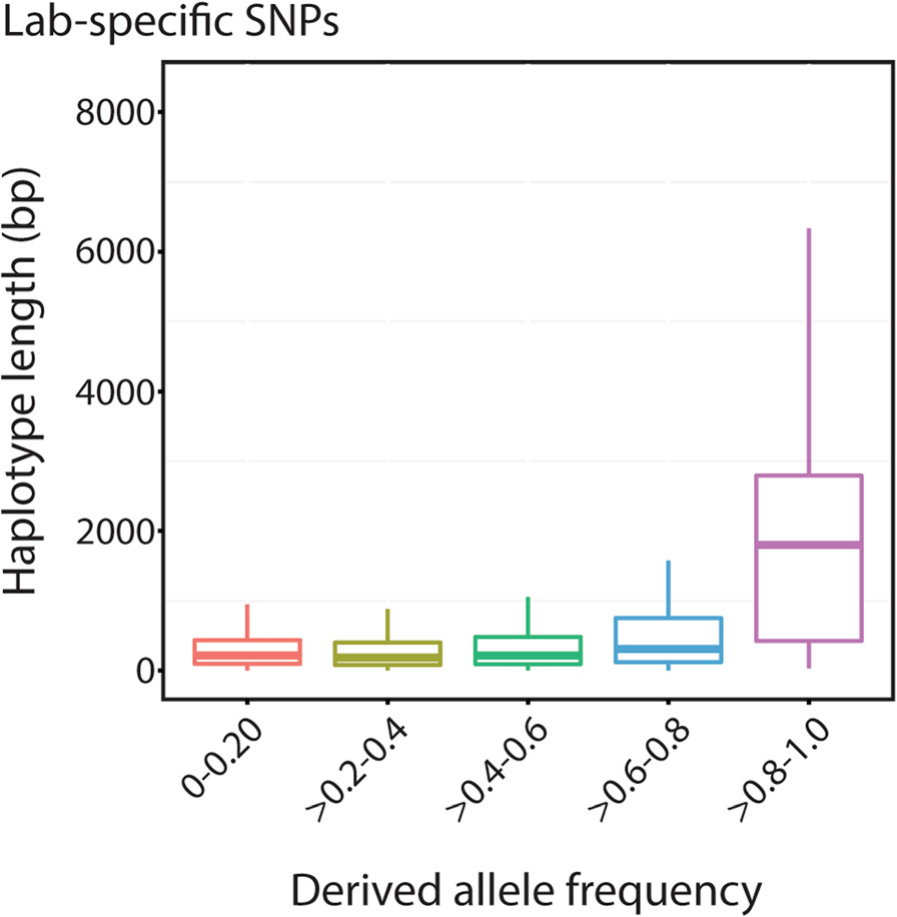
Lab-specific SNP haplotype length across frequency class. Boxplot of extended haplotype length surrounding lab-specific SNPs across derived frequency allele classes

**Fig. 5:**
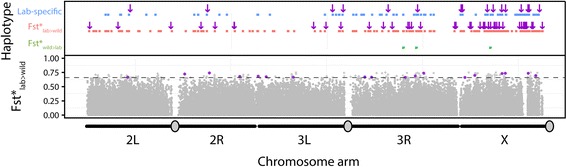
Neurogenetic enrichment of extended haplotype blocks and Fst*_wild>lab_ SNP genomic distribution. *In the top panel,* colored rectangles indicates haplotype blocks surrounding lab-differentiated SNPs (lab-specific: *blue*, Fst*_lab>wild_: red, Fst*_wild>lab_: green). Size distribution of haplotype blocks per frequency class is found in Fig. 4. Presence of known neurogenetic genes in each block is indicated by purple arrows. *In the bottom panel,* F_ST_ scan identifies derived SNPs in genic regions with higher allele frequency in the Raleigh NC population relative to the laboratory strains (Fst*_wild>lab_). Dotted line indicates cutoff for Fst*_wild>lab_ SNPs (Z-score > 2.5, above dotted line). Of the 62 Fst* SNPs found in genic regions, SNPs are significantly enriched for neurogenetic genes (*P < 0.001*). SNPs found within neurogenetic genes are shaded in *purple*

Regions of extended homozygosity present potential signals of positive selection. We observe a mean haplotype length for all lab *vs*. wild differentiated classes of SNPs (lab-specific, Fst*_lab>wild_, Fst*_wild>lab_) of 653 bp (SD = 801 bp). Among lab-specific SNPs, long outlier haplotypes were only found within the high frequency class ≥0.8 and were significantly larger than all other frequency classes (Wilcoxon rank sum test, *P* = 3.41 × 10^−4^, Fig. 4). Under a neutral model, we would expect similar haplotype lengths across all frequency classes. In addition, we have run simulations in which we choose sites at random and estimate haplotype lengths. Using 1000 replicates, the random site haplotype lengths are at least two standard errors lower than the observed 5/5 haplotype lengths. A total of 457 (lab-specific: 112, Fst*_lab>wild_: 342, Fst*_wild>lab_: 3) large haplotype block outliers (Z_hap_ >2.5) were identified ranging in length from 2,622 bp to 11,985 bp and were significantly enriched (by nearly four times the expected amount) on the X-chromosome (Fig. 5). Candidate lab-specific, Fst*_lab>wild_, and Fst*_wild>lab_ haplotype blocks contain, respectively, 135, 334, and 4 genes. Genes found within these lab-specific candidate haplotype blocks are enriched for neurogenetic gene ontology categories along with functional classes related to regulation and behavior responses (Table 1) as well as other significant functional categories (Additional file 8: Table S4). However, when normalized by gene length, significant GO category enrichments in large lab-specific haplotype blocks disappear. Within large Fst*_lab>wild_ haplotype blocks, genes are enriched for GO classes involved with axon guidance, post-embryonic system development, and regulation (Table 1), even after normalizing for gene length. No significant GO enrichment is found in the four genes contained within large Fst*_wild>lab_ haplotype blocks.

**Table 1:** Enriched gene ontologies (GO) on extended haplotype block outliers for two categories of differentiated SNPs: unique among laboratory strains (lab-specific) or higher in allele frequency in the lab (Fst*_lab>wild_)

SNP Type	GO: Biological Process	GO: ID	# of Genes in GO^a^	**P-value	Count
Lab-specific	regulation of cellular component size	32535	305	0.028412	8
Lab-specific	regulation of biological quality	65008	1994	0.038348	23
Lab-specific	generation of neurons	48699	992	0.039188	22
Lab-specific	nervous system development	7399	1744	0.040839	32
Lab-specific	neurogenesis	22008	1525	0.041256	30
Lab-specific	regulation of anatomical structure size	90066	374	0.042371	10
Lab-specific	regulation of cell size	8361	89	0.042507	6
Lab-specific	cellular component organization	16043	5855	0.042708	43
Lab-specific	proboscis extension reflex	7637	9	0.043535	3
Lab-specific	reflex	60004	9	0.043535	3
Lab-specific	cell differentiation	30154	2770	0.043641	41
Lab-specific	regulation of cell morphogenesis	22604	184	0.044224	9
Lab-specific	axon development	61564	341	0.047764	12
Lab-specific	behavioral response to nutrient	51780	10	0.048507	3
Fst*_lab>wild_	motor neuron axon guidance	8045	62	0.007739	9
Fst*_lab>wild_	system development	48731	2850	0.00912	88
Fst*_lab>wild_	post-embryonic organ development	48569	583	0.009155	29
Fst*_lab>wild_	multicellular organismal development	7275	3977	0.009225	106
Fst*_lab>wild_	biological regulation	65007	13920	0.009452	112
Fst*_lab>wild_	single-organism cellular process	44763	23874	0.014023	162
Fst*_lab>wild_	regulation of biological process	50789	13269	0.015888	106
Fst*_lab>wild_	regulation of cellular process	50794	12824	0.023125	96
Fst*_lab>wild_	single-multicellular organism process	44707	5981	0.024058	118
Fst*_lab>wild_	post-embryonic appendage morphogenesis	35120	452	0.026085	24
Fst*_lab>wild_	appendage development	48736	470	0.026896	24

^a^Number of genes from Gene Ontology (Biological Process) from FlyBase FB2015_05

**P-value calculated using Benjamini-Hochberg correction

Gene ontologies within each SNP type are ranked by *P*-value. No significant gene ontology class was found for haplotypes classified for significantly differentiated SNPs in which the derived SNP is found in lower frequency in the wild (i.e., Fst*_wild>lab_)

## Discussion

### Phenotypic and genetic differences in laboratory stocks

The captive genetic model, *Drosophila melanogaster*, reveals hallmark features consistent with domestication. Laboratory strains show significant differences in behavior from their relatively recently isolated wild progenitors (Additional file 1: Figure S1). Previous studies comparing lab and wild-caught lines of *D. melanogaster* also report differences in egg and larval survival [31] and life history traits such as pre-adult development, early fecundity, and remating frequency [32, 33]. These traits comprise a suite of behavioral, physiological, and reproductive characters [34] that have converged across multiple strains evolving independently under similar laboratory conditions. Furthermore, it previously has been demonstrated that these traits can quickly evolve significant differences in as little as 8–10 generations [35].

It is paradoxical that domesticates, typically derived from small founder populations and maintained at very low effective population sizes, can effectively adapt to human conditions. From a population genetics perspective, we may expect the opposite: that small captive populations and lower N_e_ propagate the segregation and eventual fixation of deleterious mutations, thus, imposing a potentially large mutational burden on laboratory strains [36, 37]. Our evolutionary parameter analysis reveals such drift-like signatures at the low end of the site frequency spectrum. Estimates of Grantham distances, R/S ratios, codon positional fraction, and codon bias support a general genome-wide reduction in selection on low-frequency derived alleles in the lab (Fig. 3) where mildly deleterious alleles may persist for longer periods of time [38]. With a decrease in effective population size across each strain, inbreeding and drift dominate, which in turn, can quickly fix allelic and associated phenotypic changes across successive generations of captivity. Short-term isolation studies in Drosophila have shown similar rapid changes across a variety of phenotypes [35, 39] with reductions in performance levels [40]). Thus, the accumulation and fixation of mildly deleterious alleles, particularly on larger neurogenetic genes (see below), and subsequent inbreeding depression [41, 42] may promote both the rapid nature of domestication and its associated convergence of common behavioral traits. These docile and non-aggressive traits may alternatively be described as “lethargic” (at least relative to their wild-caught conspecifics), a term commonly applied to inbred, and often sickly, Drosophila stocks.

When did these changes occur? Our results suggest that these changes towards a domesticated phenotype likely began very early on based on available standing genetic variation of the progenitor population. While heterozygosity in each contemporary isofemale lab strain is virtually zero (data not shown), as a whole, these five lab strains collectively only harbor a two-fold genome-wide decrease in nucleotide diversity, Θ_lab_, relative to a large contemporary population from Raleigh NC (Additional file 7: Figure S4). This diversity estimate primarily reflects the amount of genetic variation captured in each of the five lines from an ancestral population(s). The five lab strains share ~800,000 derived SNPs with extant global populations (a total of ~10 million non-singleton *D. melanogaster* SNPs pass our data filters) indicating that laboratory stocks collectively extracted a significant fraction *D. melanogaster* genetic variation at their time of capture.

The importance of ancestral standing genetic variation can also be seen in the 17,250 lab-specific SNPs. In theory, these SNPs represent any of the following: i) *de novo* mutations that arose in the lab, ii) genetic variation that previously existed in an extinct North American population, or iii) a subset of genetic variation that has been completely lost in extant global populations. We estimate that only a small fraction of these SNPs can be generated *de novo* (3.5 × 10^−9^ mutations/bp/generation x 120 × 10^6^ bp x 20 generations/yr x ~75 years x 5 strains ≈ 3,150 lab-specific SNPs [43]). The remainder of the lab-specific SNPs was probably lost in extant wild populations during the last century. Strong evidence supports a recent global sweep in *D. melanogaster* that dramatically reduced species-wide genetic variation after these particular lab strains were collected [44]. Thus, from the large pool of available genetic variation from their North American progenitor populations, Canton-S and Oregon-R likely experienced similar selection pressures on common genetic variants (see below) during the earliest generations of lab domestication [45]. A phylogenetic analysis of shared lab/wild SNPs also supports a distinct origin of all lab strains (Additional file 6: Figure S3). However, whether the lab strain monophyly is the result of an extinct progenitor population or the loss of global variation is unknown. The inclusion of more sequenced lab strains may differentiate between these two hypotheses.

### Adaptation in the lab

*Caenorhabditis elegans*, like *D. melanogaster*, has been lab-cultured for over 50 years, and harbors pronounced differences in longevity and fertility when compared to wild isolates [46]. Genome sequencing in the nematode identified SNPs differentiated between wild and long-term laboratory strains enriched for cell cycle and metabolic/growth genes [13]. Their results suggest the presence of strong selection early in nematode domestication for optimal growth under rich nutrient conditions similar to the significant GO term, “behavioral response to nutrients”, found among lab-specific SNPs in fruit flies (Table 1). Laboratory mice have similarly been shown to converge certain phenotypes including melatonin deficiency [15], and a lack of aggression and tameness [47, 48]. The results from our evolutionary parameter analysis, when limited to lab-specific SNPs in the mid- to high frequency range, support that common phenotypic signals of domestication observed in the captive fruit fly have been strongly shaped by selection. These derived SNPs, found in the majority of lab strains, show similar evolutionary patterns to high frequency SNPs from the wild and not to neutral expectations, unlike low-frequency SNPs (Fig. 3). The laboratory setting presents an immediate change in the fitness landscape, permitting rapid and significant changes in phenotype that would be detrimental to their fitness in the wild, across relatively few generations [35, 36].

Inadvertent human habituation and unintentional conditioning may be the primary selective agent for such known differences among lab strains as faster development and reproductive time [13, 14]. For instance, flies that rarely escape the bottle or benchtop may be selected due to human carelessness while fly stocks are transferred to new vials/bottles, or “flipped”. Our behavioral results support such a convergent shift towards less active and responsive flies (Additional file 1: Figure S1). From our genomic analysis, we find that neurogenetic genes, involved in such biological processes as neurogenesis and axon development, are enriched in extended haplotype blocks common to differentiated SNPs (both lab-specific and highly differentiated Fst*) found at high allele frequencies (≥0.8), with an overrepresentation of fixations on the X-chromosome. These genes affect locomotion and visual cues suggesting lab selection on genes involved in behavioral responses.

While an excess of long extended haplotypes on high frequency lab-specific SNPs support an adaptive shift towards domesticated phenotypes, a relaxation of selection on certain loci involved in behavior may have co-occurred in the lab. Conditions in the laboratory are often optimized for growth and reproduction, reducing the natural ability of flies to escape predators or compete for food and mates. Thus, a relaxation of selection on activity levels, aggressiveness, and responsiveness, critical in the wild, may also drive the behavioral differences that converged across lab strains. Characters involved in mating, driven by the sparsity of mates in the vicinity, is a key difference between domestic and wild species [49]. Our behavioral results, showing a reduction in interactive activity in lab flies, is consistent with this hypothesis. In addition, our GO analysis of shared Fst*_wild>lab_ SNPs, in which the derived allele is more frequent in the Raleigh NC population than lab strains, finds a significant enrichment of the neurogenetic functional class, even when corrected for gene size. These SNPs are found in different genes than the SNPs harbored in long extended haplotype blocks, suggesting an extensive cache of genes involved in behavioral differences between flies reared in the lab and those found in the wild. A similar decrease in behavioral activity was observed in lab strains of mice [50], with backcrossing to wild mice isolates allowing them to regain these previously lost behavioral functions [47].

The use of inbred laboratory strains of *D. melanogaster* presented unique analytical challenges that differ from other domesticated studies. Due to initial and recurrent inbreeding and the lack of an interbreeding population, laboratory strains violate most population genetic models used to infer selection. In this study, the site frequency spectrum is only applied across isolated lab strains as a framework to bin our observed data and could not be used to infer population genetic parameters. Despite these difficulties, our results reveal an interplay of drift and selection at work in the lab. First, we find genome-wide levels of selective constraints in the lab that are significantly lower than a sampled North American population. This pattern is likely caused by low effective population sizes in bottles that promote the accumulation of mildly deleterious mutations under drift-like conditions, which we also observe in low frequency alleles. Second, we observe derived SNPs that are highly differentiated between the lab and a North American population to be significantly enriched in neurogenetic genes, suggesting a differential fitness landscape in behavior. This functional enrichment takes into account the number of genes in each functional class as well as their size. Third, we find signatures of positive selection on extended haplotypes in both lab-specific and highly differentiated SNPs. These, too, are significantly enriched in neurogenetic genes. Fourth, there’s an enrichment of these changes on the X-chromosome (Fig. 2; Fig. 5; Additional file 2: Table S1). The preferential role of the X-chromosome is seen in marked differences in the site frequency spectrum between the X-chromosome and autosomes (Fig. 2b) and in the enrichment of long haplotype blocks on the X-chromosome (Fig. 4). Since many of these long X-linked haplotype blocks are fixed in all five lab strains, the fixation of hemizygous loci likely occurred early in fly domestication.

### Preferential role for neurogenetic genes

Our results suggest a central role for neurogenetic genes in domestication. Lab-specific SNPs found in the majority of lab strains are strongly enriched for this functional class, as are highly differentiated SNPs found in high frequency in the wild (Fst*_wild>lab_ SNPs; Additional file 4: Table S3). Large outlier haplotype blocks also contain an overrepresentation of neurogenetic genes (Fig. 5; Additional file 8: Table S4). In most fly labs, inadvertent selection is inevitable: more active, reactive, and sensory-prone flies (and their alleles) have a higher probability of escaping during routine stock transfers. Hence, fly researchers may have unconsciously selected for lethargic flies over thousands of generations in the lab. Selected genes, enriched for sensory functions in eye photoreceptors and peripheral nervous system, can explain these behavioral shifts seen in lab strains. The significance of neurogenetic genes in changing activity and response behaviors across a relatively short evolutionary time period may also relate to how behaviors involved in premating isolation [51–53] can swiftly and easily develop in a population by drift and selection.

Recently, Wilkins et al. [54] proposed a general hypothesis to explain the convergence of various phenotypic traits that differentiate mammalian domesticates from their wild progenitors. These traits are collectively known as the “domestication syndrome” [55, 56] and, in mammals, include such morphological modifications as depigmentation, facial skeletal, and floppy ears as well as behavioral shifts towards docility and tameness [57]. Wilkins et al. [54] argue that a developmental deficit in neural crest genes can generate each of these differences, thus, explaining the commonality of these traits across domesticated mammals. Our results extend the behavioral component of the domestication syndrome to non-vertebrates but through a more general genomic mechanism based on the predominance of mutations on neurogenetic genes affecting overall locomotion and activity. In Drosophila, genes from this ontological category are among the largest in gene number and gene size, providing a large mutational target for rapid behavioral change (Additional file 9: Figure S5). Currently, 1,708 out of 17,716 genes are characterized as “neurogenetic” (according to FlyBase R6.05), and 24 % of known fly genes are expressed in the brain and nervous system [58]. We propose that a large mutational target [85] of neurogenetic genes can explain the rapid evolution of behavior in animal taxa., These neurogenomic loci collectively provide a large genomic substrate for variation to accumulate, and then selection and drift to act, to quickly transform behavior within a relatively short time frame.

## Conclusions

Tameness and docility are hallmark features of domestication and the product of artificial selection by breeders. Our results challenge the traditional notion that relatively submissive laboratory animals are solely the product of cumulating deleterious mutations and demonstrate how unconscious selection for human-favored traits plays an important role in driving rapid phenotypic change in the lab. Selection on a large pool of available genetic variation during the early stages of fly domestication, followed by strong and recurrent inbreeding, allow for the successive roles of adaptation and drift in shaping the genetic architecture of domesticated phenotypic traits in a bottle. Our study finds that the genes and phenotypes in fruit fly domestication are enriched in, respectively, neurogenetic and behavioral function, providing a starting point to decode the genomic basis of domestication and promoting its study in genetic model systems such as Drosophila. A detailed mapping of these genes and their SNPs to specific behaviors will not only be informative about the selective pressures that we have inadvertently applied to our immediate biotic environment, but may also provide new general insight on the divergence and isolation of populations.

## Methods

### Behavioral assays

Locomotory assays were performed separately on five lines of adult *D. melanogaster* (6–8 days post-eclosion), each of North American origin. Three lines (Canton-S, w^1118^, and Oregon-R) represent common laboratory stocks originally extracted from nature at least seventy years ago (Fig. 1). Two wild-caught lines were collected from Linvilla PA and Lancaster MA and reared under normal laboratory conditions for less than one year without specific selective regimes (courtesy of the Schmidt lab, University of Pennsylvania). Stocks were maintained in the laboratory at ~24 C, at ~40 % relative humidity, kept in standard 250 ml bottles on Lewis food medium [59], and exposed to a 12 h light–dark cycle. Prior to behavioral assays, flies are anesthetized with light CO_2_ sedation (<15 s) for transfer and identification, and allowed to acclimatize in the arena setting for 30 min, post-sedation. Assays are conducted in Delrin arenas (McMaster-Carr) following specifications outlined in Simon and Dickinson [60] to optimize mobility and provide an effective environment for automated tracking. To prevent locomotion on the ceilings, glass covers are coated with Sigmacote (Sigma Aldrich). Between assays, arenas are rinsed with ethanol and allowed to dry for a minimum of 15 min to ensure no residues remain from previous behavioral experiments.

Assays are conducted during active afternoon periods across successive days. Each line is recorded using three independent replicates per line tested on different days and randomized to reduce experimental bias. Fly activity, post-acclimation, is recorded for 30 min. Individual and interactive activities are tracked using CTRAX and MATLAB [61]. Errors in the initial tracking are corrected using CTRAX’s *Fix errors* scripts. All output measurements are analyzed using MATLAB and statistics implemented using custom R scripts.

### Genomic data sources

Whole genomic sequencing (125 bp paired-end) reads from three separate Oregon-R lines were downloaded from NCBI (SRX671605, SRX671606, SRX671607). Illumina 150 bp paired-end reads from Canton-S and w^1118^ were obtained from the Hawley lab (Stowers Institute). Reference assemblies were generated by aligning filtered reads against the *D. melanogaster* genome following methods described by Lack et al. [62], an assembly pipeline that adds an intermediate realignment step for the purpose of aligning reads around insertion and deletion sites. Briefly, paired-end reads were aligned using BWA v0.7.12 [63] against the complete *D. melanogaster* reference genome (*Dmel* Release 5) obtained from FlyBase (flybase.org). Post-alignment files were transformed using SAMtools [64] and Picard v1.79 (broadinstitute.github.io/picard/). Bases are filtered for a minimum quality score of 30 and a minimum read depth of 15x from VCF files generated by the Genome Analysis Toolkit [65]. Additional file 10: Table S5 includes a brief summary of the raw data.

To identify mutational states in the lab, 516 full genome assemblies from natural populations of *D. melanogaster* were downloaded from the Drosophila Genome Nexus [62] representing 23 countries from Africa, Europe, and North America [20, 66, 67]. Genome-genome alignment of *D. simulans* R2 assembly [68] against the *D. melanogaster* R5 assembly was performed using Progressive Mauve [69] using default parameters. To validate the quality of our alignment, the average number of nucleotide substitutions (Dxy; [70]) was estimated for 100 kb non-overlapping windows (Additional file 11: Figure S6) and genome-wide patterns compared to previous literature [71].

### Genomic filters and annotations

All genomic analyses were restricted to euchromatic chromosome arms (2L, 2R, 3L, 3R, X). To minimize sampling biases, the combined dataset was subjected to coverage filters for missing data. For a particular site to be used, a minimum 75 % of the laboratory strains (*n* > 3) and a minimum of 75 % of the population samples (*n* ≥ 388) must contain a non-ambiguous nucleotide, with no more than two alleles present (i.e., only monoallelic and diallelic sites were included). SNPs are identified across all filtered base pairs, with singletons from global populations excluded to conservatively reduce the effects of sequencing error from low-coverage global samples. Data were also filtered for the presence of a *D. simulans* allele to infer ancestral state*.* After filtering for data quality, coverage, and ancestral state, 98,442,787 eligible sites (Additional file 12: Table S6) were used to identify SNPs differentiated between the lab and wild.

We identify several types of derived mutations: SNPs unique to lab strains and SNPs significantly differentiated between lab stains and the wild. Derived SNPs uniquely found in labs (i.e., the lab strain(s) possess a base neither present in known global populations of *D. melanogaster* nor *D. simulans*) are classified as “lab-specific” SNPs. Highly differentiated SNPs, often shared across both lab and wild samples, were identified via Hudson’s F_ST_ estimator [72, 73] with F_ST_ scores Z-transformed as follows: Z-F_ST_ = (F_ST_ - μF_ST_)/σF_ST_. SNPs with F_ST_ estimates harboring a Z-score > 2.5 were considered highly differentiated (Fst*) SNPs. Fst* SNPs are further classified as either: i) “Fst*_lab>wild_” if the derived allele is found at a higher frequency in the lab, or ii) “Fst*_wild>lab_” if the derived allele is higher in frequency in the wild. Both lab-specific and Fst* SNPs can be further categorized as polymorphic (1/5,…4/5) or fixed (5/5, or 4/4 in the case of missing data) with respect to their frequency among the five sequenced lab strains.

SNPs are annotated by genomic location (e.g., genic *vs*. inter-genic) using FlyBase R5.9. SNPs located within a gene model, represented by their longest transcript, are further classified according to their annotated position within a gene model (5’UTR, exon, intron, 3’UTR), and their codon position (C1, C2, C3) if found within an exon. SNPs found within exonic regions are also classified as non-synonymous or synonymous. The relative fitness of each amino acid substitution is estimated using a Grantham score [74], which evaluates biochemical dissimilarity (based on polarity, amino acid size, and side chain composition) between ancestral and derived states, with lower Grantham scores indicating a greater biochemical similarity. Synonymous codon shifts are categorized into four separate classes (P2P, preferred codon > preferred codon; P2N, preferred codon > non-preferred codon; N2P, non-preferred codon > preferred codon; N2N, non-preferred codon > non-preferred codon) according to the classification of Vicario et al. [75].

### Detecting selective signals

To determine whether selection is acting in the lab, we compared evolutionary patterns of coding region variation in lab-specific SNPs against: i) a North American population comprising of 205 DGRP genomes from Raleigh NC [28, 62] and ii) neutral expectations. Since negative and positive selection differentially affects the site frequency spectrum, we bin these differentiated SNPs according to their shared frequency among lab strains: with five strains, the site frequency spectrum is divided into fifths.

To generate neutrally simulated data, *n* mutations, based on the number of SNPs found in the CDS of lab strains, are randomly assigned to *D. melanogaster* coding regions. SNPs within CDS regions are labeled according to their codon position (C1, C2, C3) and classified as a non-synonymous or synonymous substitution with synonymous SNPs classified as preferred or non-preferred codons. A simple model of equal probability of changing any position within the codon to another nucleotide is applied. 1000 simulations are performed. After binning these simulated data into the five allele frequency classes, we estimate basic evolutionary parameters including mean Grantham score of amino acid substitutions, proportion of non-synonymous SNPs, fraction of polymorphisms in 1^st^ codon position, and shifts in codon preference. Wilcoxon rank-sum tests [76] are used to compare these neutral estimates, as well as those from the North Carolina population (*n* = 205), against parameter estimates from the lab strains (*n* = 5).

Recent domestication studies have surveyed genomic regions for significantly reduced heterozygosity [8, 77, 78] to identify selectively swept candidate genes. However, heterozygosity is rare, if not absent, in isogenic strains of *D. melanogaster* (data not shown). For each SNP, we estimate the mean population (i.e., laboratory) haplotype length conditioned on frequency class. Haplotype length analyses are performed using custom perl scripts allowing for non-congruent haplotypes to extend from each lab-specific SNP. Due to the lower sequencing coverage of lab strains, a maximum of one individual per site is permitted to have missing data. Large outlier haplotype blocks are identified by a Z-hap score > 2.5.

### Phylogenetic and functional enrichment analyses

To understand the topological relationship among lab strains, neighbor-joining trees [79] are generated using p-distance [70] and bootstrapped 1,000 times [80] for laboratory-specific and high F_st_* (Fst*_lab>wild_ + Fst*_wild>lab_) SNP sets. To evaluate the topologies between lab strains and extant populations, 100,000 SNPs that are shared in lab and nature were randomly chosen for NJ tree analysis and bootstrapped 1,000 times using MEGA6 [81]. Overrepresented gene ontologies for differentiated SNPs are identified using DAVID [82] and FlyMine [83]. Gene sets are weighted according to the size of their categories and a False Discovery Rate (FDR) is used to correct for the deployment of multiple tests. For selected enrichment analyses, gene lengths were used to normalize the potential impact of genes from certain GO categories covering a disproportionate fraction of the genome. Gene annotation data for tissue specificity and ontogenetic stages are characterized using FlyAtlas [84].

## Additional files

Additional file 1: Figure S1. Differences in activity between laboratory and wild-caught Drosophila. (A) Mean fraction of time spent moving per angular velocity bin (radians/sec) for laboratory (red) and wild-caught (green) flies. Shading indicates standard error across replicates (B) Mean fraction of time spent moving per forward velocity bin. Laboratory flies spend significantly greater proportion of time at lower angular and forward velocity then their wild conspecifics (P-value < 0.05, Mann–Whitney U Test). (C) Mean fraction of time per fly spent walking during a 30 min assay. (D) Fraction of time per fly spent stationary (velocity = 0 m/s). (E-F) Relationship of distance to nearest neighboring fly and its velocity. Heatmap colors denote velocity gradient for wild (E) and lab (F) flies. Wild-caught flies are generally more active with greater velocity when in closer proximity to other flies. (TIF 32946 kb) Additional file 2: Table S1. SNP characterization. Number and genomic location of laboratory-specific, high-F_ST_/high lab (Fst*_lab>wild_), and high-F_ST_/low lab SNPs (Fst*_wild>lab_). Expected proportions based on genic fractions garnered from FlyBase *Dmel* R5.9. Random simulations capped using the number of laboratory-specific SNPs. (XLSX 43 kb) Additional file 3: Table S2. Gene characterization. Characterization of genes containing highly differentiated lab SNPs. (XLSX 244 kb) Additional file 4: Table S3. Gene ontology. Significant Gene Ontology (GO) classes for genes containing highly differentiated SNPs. Enrichments were normalized by gene lengths among gene ontology categories. Significance adjusted for multiple tests via Benjamini-Hochberg correction (Benjamini and Hochberg 1995). (XLSX 48 kb) Additional file 5: Figure S2. Tissue expression. Distribution of tissue expression for coding region SNPs highly differentiated between lab strains and wild-caught lines. (A) Lab-specific SNPs, (B) Fst*_lab>wild_ SNPs, (C) Fst*_wild>lab_ SNPs. (TIF 10759 kb) Additional file 6: Figure S3. Neighbor-joining trees. Phylogenetic trees for (A) lab-specific SNPs (B) highly differentiated (Fst*) SNPs, and (C) random 100,000 polymorphic sites. Bootstrap values for 1000 replicates are placed at each node. Numeric node labels represent individuals from lab strains (1–5), Raleigh, NC (6–210), France (211–219), and Africa (220–521). Location of the laboratory strains are highlighted using red branches. (TIF 38440 kb) Additional file 7: Figure S4 Genome-wide nucleotide diversity. Genome-wide distribution of nucleotide diversity (θ_s_) across 50,000 bp non-overlapping windows in (A) a Raleigh NC population and (B) all lab strains. Centromeres are denoted as ovals. (TIF 15720 kb) Additional file 8: Table S4. Large extended haplotype gene characterization. Characterization of genes found within large extended haplotype blocks surrounding SNPs (Lab-specific, Fst*_lab>wild_, and Fst*_wild>lab_). (XLSX 29 kb) Additional file 9: Figure S5. Genomic coverage of functional classes. (A) Genes are functionally classified by gene ontology and may overlap multiple classes. Neural functional class is highlighted in blue. (B) Comparison between average size of neural *vs*. non-neural functional classes across gene regions. (TIF 7433 kb) Additional file 10: Table S5. Summary of sequenced reads, strain origin, and distribution of lab-specific SNPs for each assembled laboratory strain. Counts within parentheses denote the number of lab-specific SNPs found among the five laboratory strains for each of the frequency classes, 1/5, 2/5, 3/5, 4/5, and 5/5. Each assembly used paired-end Illumina reads. All reads were filtered for quality and assembled against the *D. melanogaster* R5 genome (see Materials and Methods for further details). (XLSX 8 kb) Additional file 11: Figure S6. Genome-wide nucleotide substitution (Dxy) plot for *D. simulans* and *D. melanogaster*. Dxy was calculated using 100,000 bp non-overlapping windows. Ovals denote centromeres. (TIF 14250 kb) Additional file 12: Table S6. Base call quality control. Genome quality control filters use sequence coverage and the presence of a *D. simulans* R2 base call across chromosome arms. The number of lab-specific SNPs are indicated per chromosome arm. (XLSX 42 kb)

## Abbreviations

bp: base pairs
CDS: coding sequence
Fst*: highly differentiated Fst
Fst*_lab>wild_: high Fst with larger allele frequency in lab
Fst*_wild>lab_: high Fst with larger allele frequency in wild
GO: Gene Ontology
CNS: Central Nervous System
R/S: ratio of replacement to synonymous substitutions
C1/C2+C3: ratio of the first position codon to 2^nd^ and 3^rd^ position codons
Θ: nucleotide diversity
SNP: single nucleotide polymorphism
Z_hap_: Z-score haplotype
SD: standard deviation
NC: North Carolina
NCBI: National Center for Biotechnology Information
UTR: untranslated region
P2N (P2P: N2P, N2N), preferred to non-preferred codons
DGRP: Drosophila Genome Reference Panel
