# Supplementary figures and images for: Genomic signatures of domestication on neurogenetic genes in Drosophila melanogaster
Source: BMC Evol Biol. 2016 Jan 5;16:6. doi: 10.1186/s12862-015-0580-1 (PMC4700609; doi:10.1186/s12862-015-0580-1)

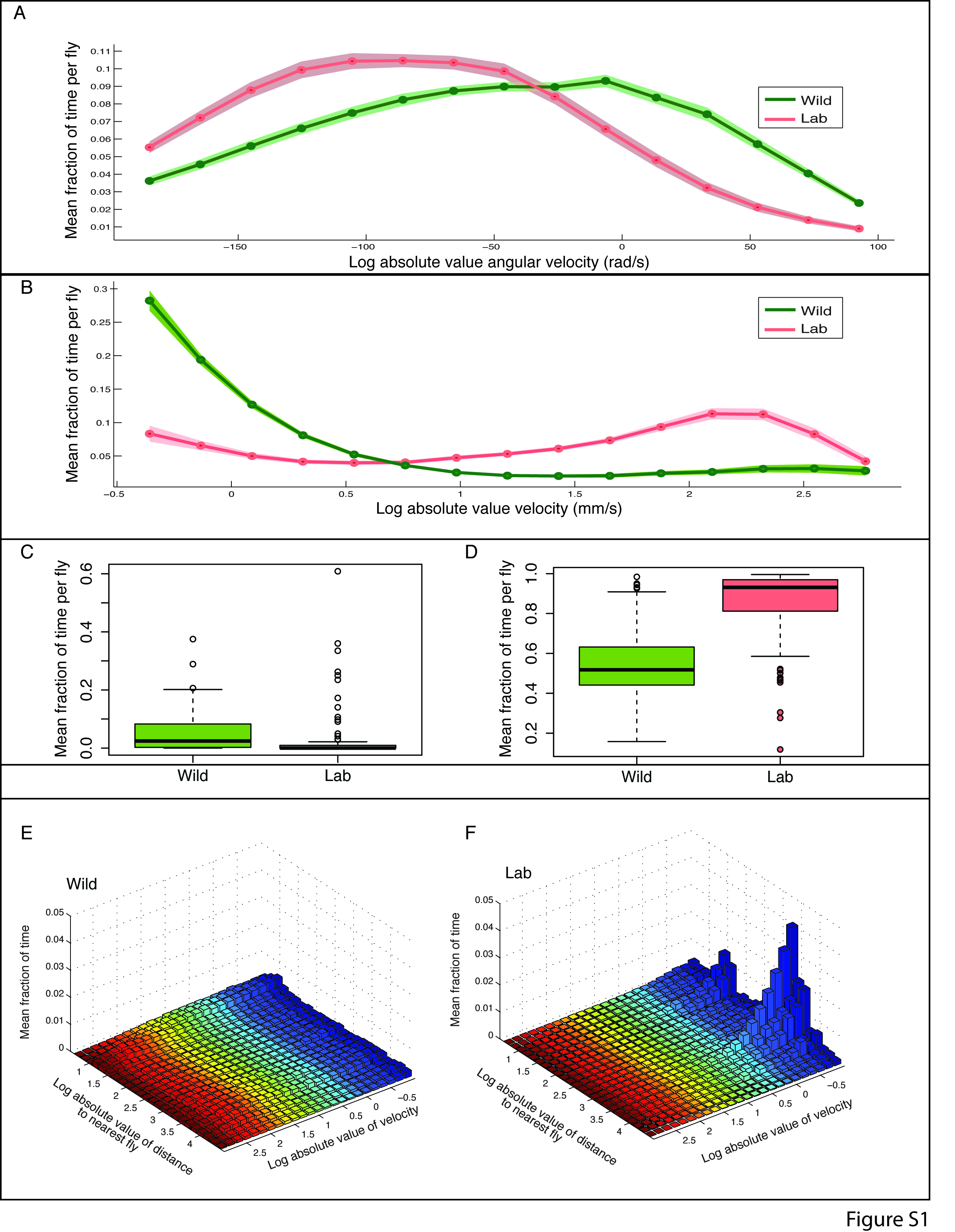

Supplement: Additional file 1: Figure S1. — Differences in activity between laboratory and wild-caught Drosophila. (A) Mean fraction of time spent moving per angular velocity bin (radians/sec) for laboratory (red) and wild-caught (green) flies. Shading indicates standard error across replicates (B) Mean fraction of time spent moving per forward velocity bin. Laboratory flies spend significantly greater proportion of time at lower angular and forward velocity then their wild conspecifics (P-value < 0.05, Mann–Whitney U Test). (C) Mean fraction of time per fly spent walking during a 30 min assay. (D) Fraction of time per fly spent stationary (velocity = 0 m/s). (E-F) Relationship of distance to nearest neighboring fly and its velocity. Heatmap colors denote velocity gradient for wild (E) and lab (F) flies. Wild-caught flies are generally more active with greater velocity when in closer proximity to other flies. (TIF 32946 kb) [file 12862_2015_580_MOESM1_ESM.tif]

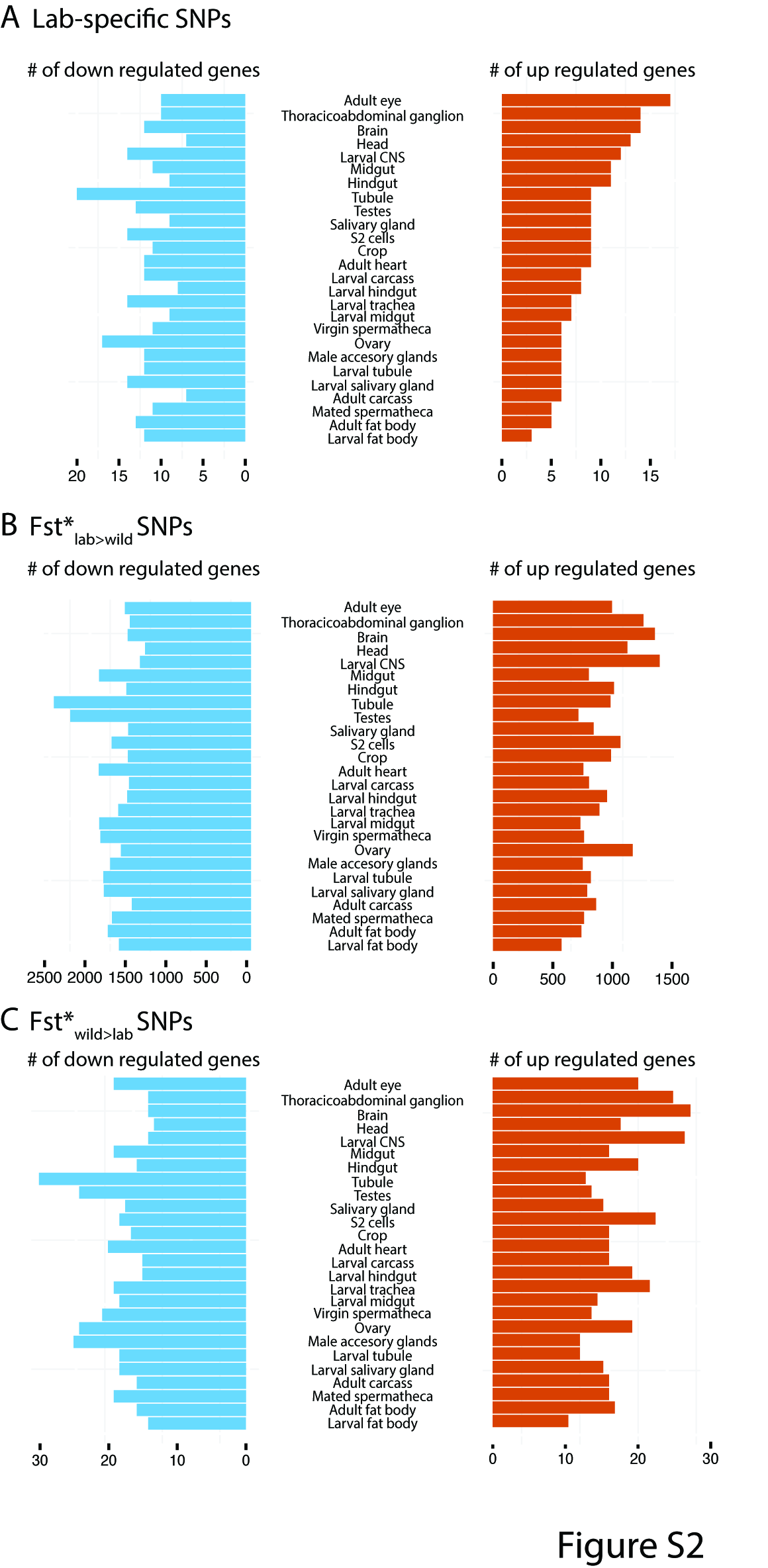

Supplement: Additional file 5: Figure S2. — Tissue expression. Distribution of tissue expression for coding region SNPs highly differentiated between lab strains and wild-caught lines. (A) Lab-specific SNPs, (B) Fst*lab>wild SNPs, (C) Fst*wild>lab SNPs. (TIF 10759 kb) [file 12862_2015_580_MOESM5_ESM.tif]

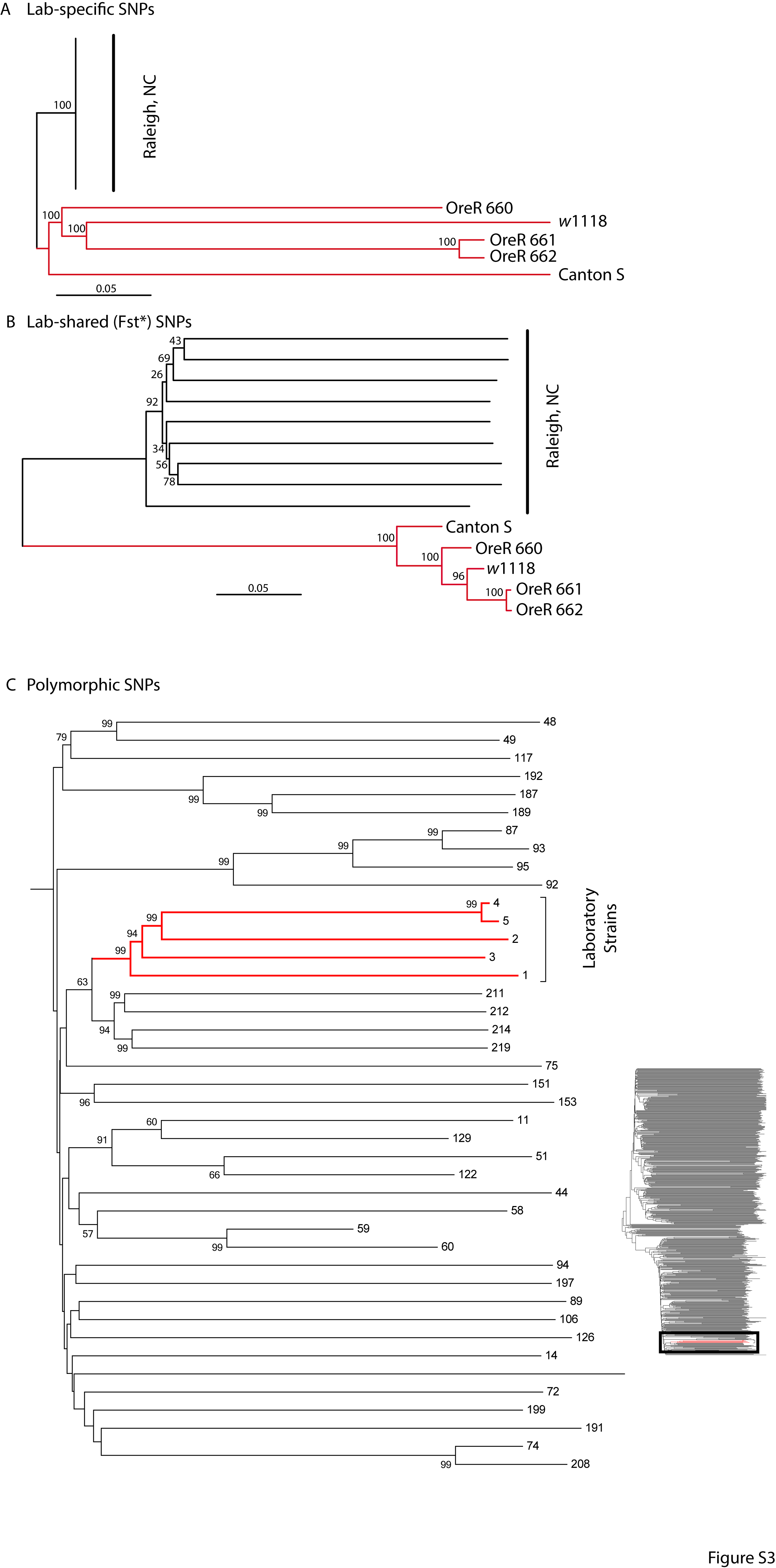

Supplement: Additional file 6: Figure S3. — Neighbor-joining trees. Phylogenetic trees for (A) lab-specific SNPs (B) highly differentiated (Fst*) SNPs, and (C) random 100,000 polymorphic sites. Bootstrap values for 1000 replicates are placed at each node. Numeric node labels represent individuals from lab strains (1–5), Raleigh, NC (6–210), France (211–219), and Africa (220–521). Location of the laboratory strains are highlighted using red branches. (TIF 38440 kb) [file 12862_2015_580_MOESM6_ESM.tif]

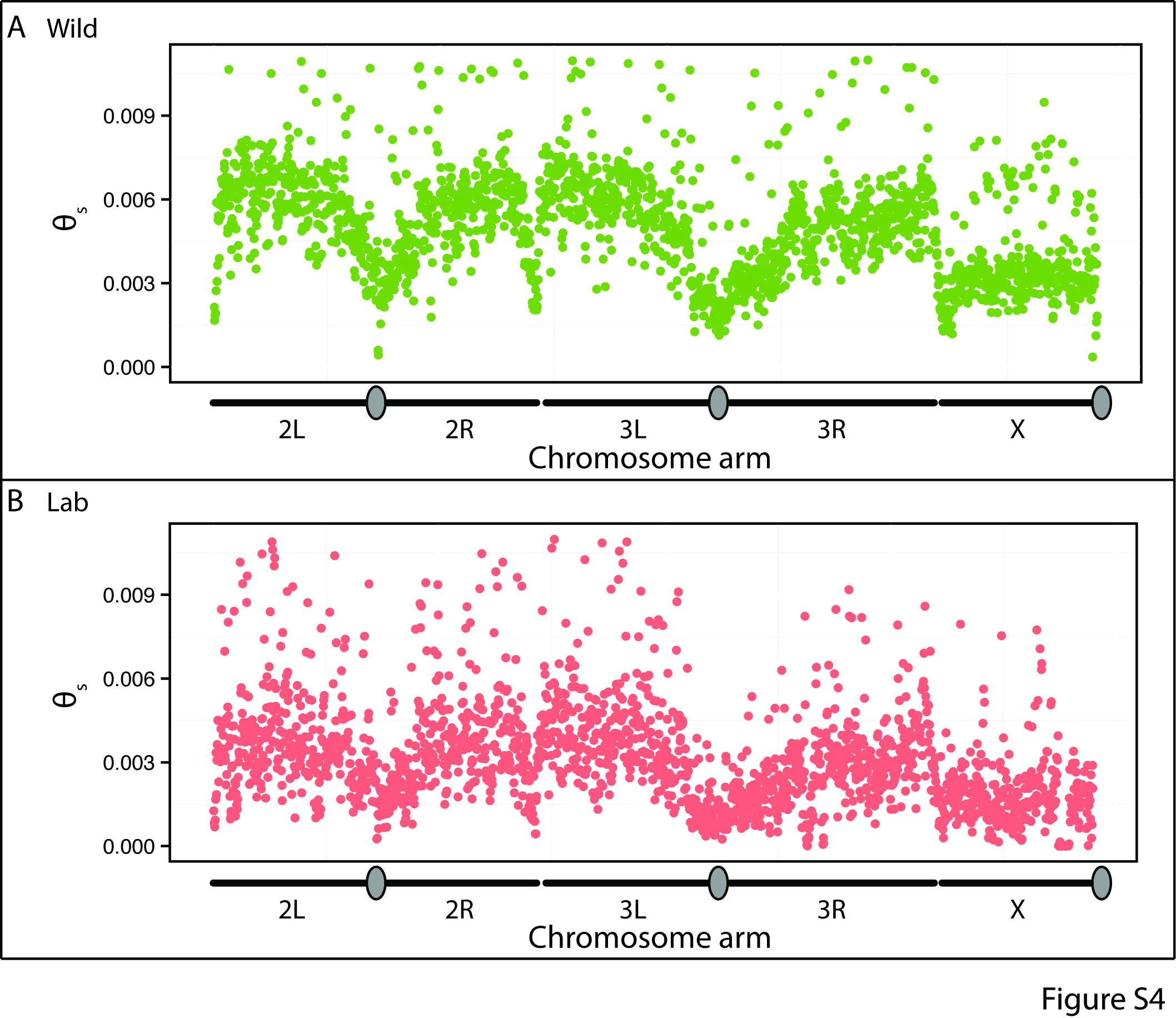

Supplement: Additional file 7: Figure S4 — Genome-wide nucleotide diversity. Genome-wide distribution of nucleotide diversity (θs) across 50,000 bp non-overlapping windows in (A) a Raleigh NC population and (B) all lab strains. Centromeres are denoted as ovals. (TIF 15720 kb) [file 12862_2015_580_MOESM7_ESM.tif]

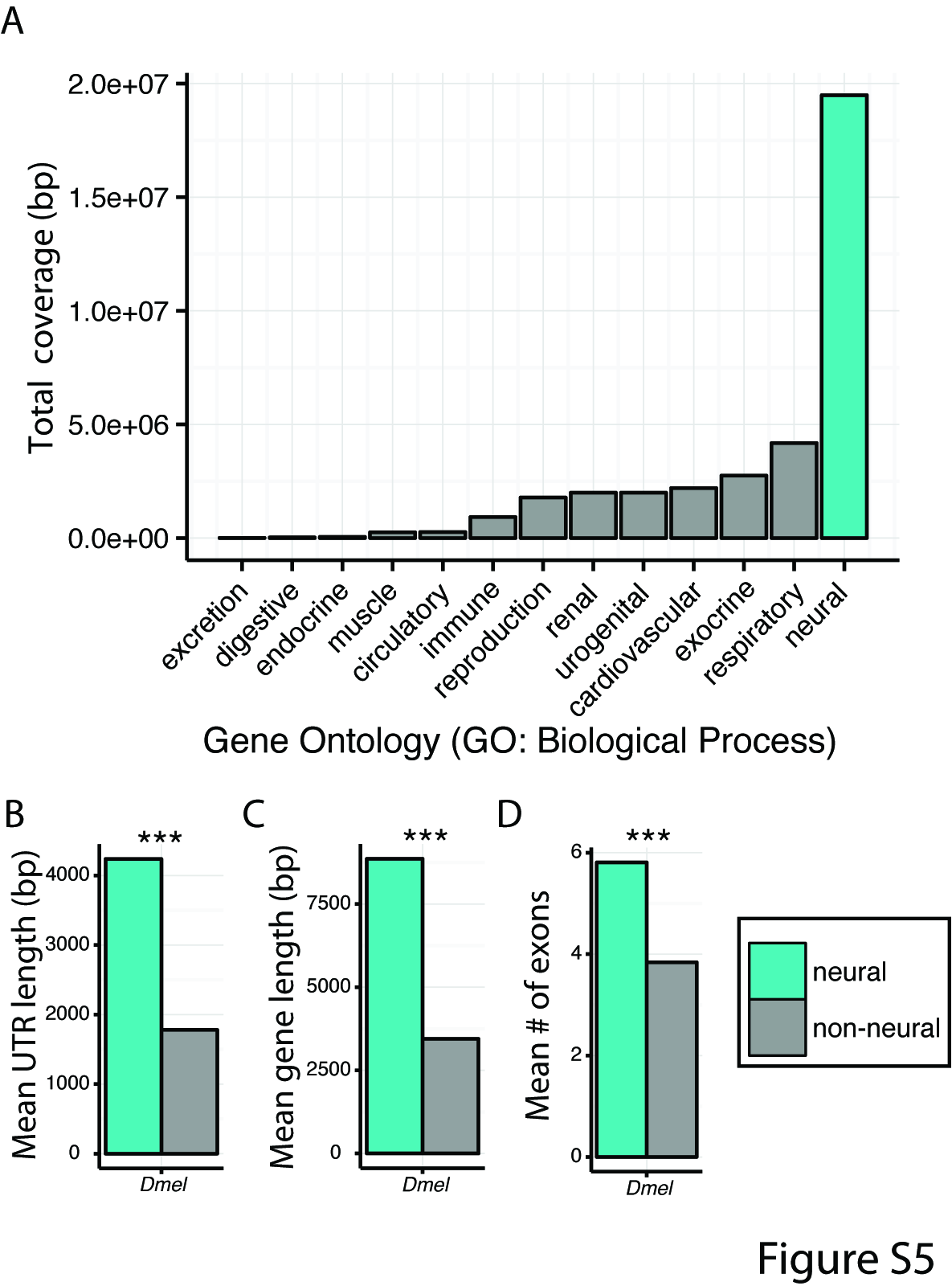

Supplement: Additional file 9: Figure S5. — Genomic coverage of functional classes. (A) Genes are functionally classified by gene ontology and may overlap multiple classes. Neural functional class is highlighted in blue. (B) Comparison between average size of neural vs. non-neural functional classes across gene regions. (TIF 7433 kb) [file 12862_2015_580_MOESM9_ESM.tif]

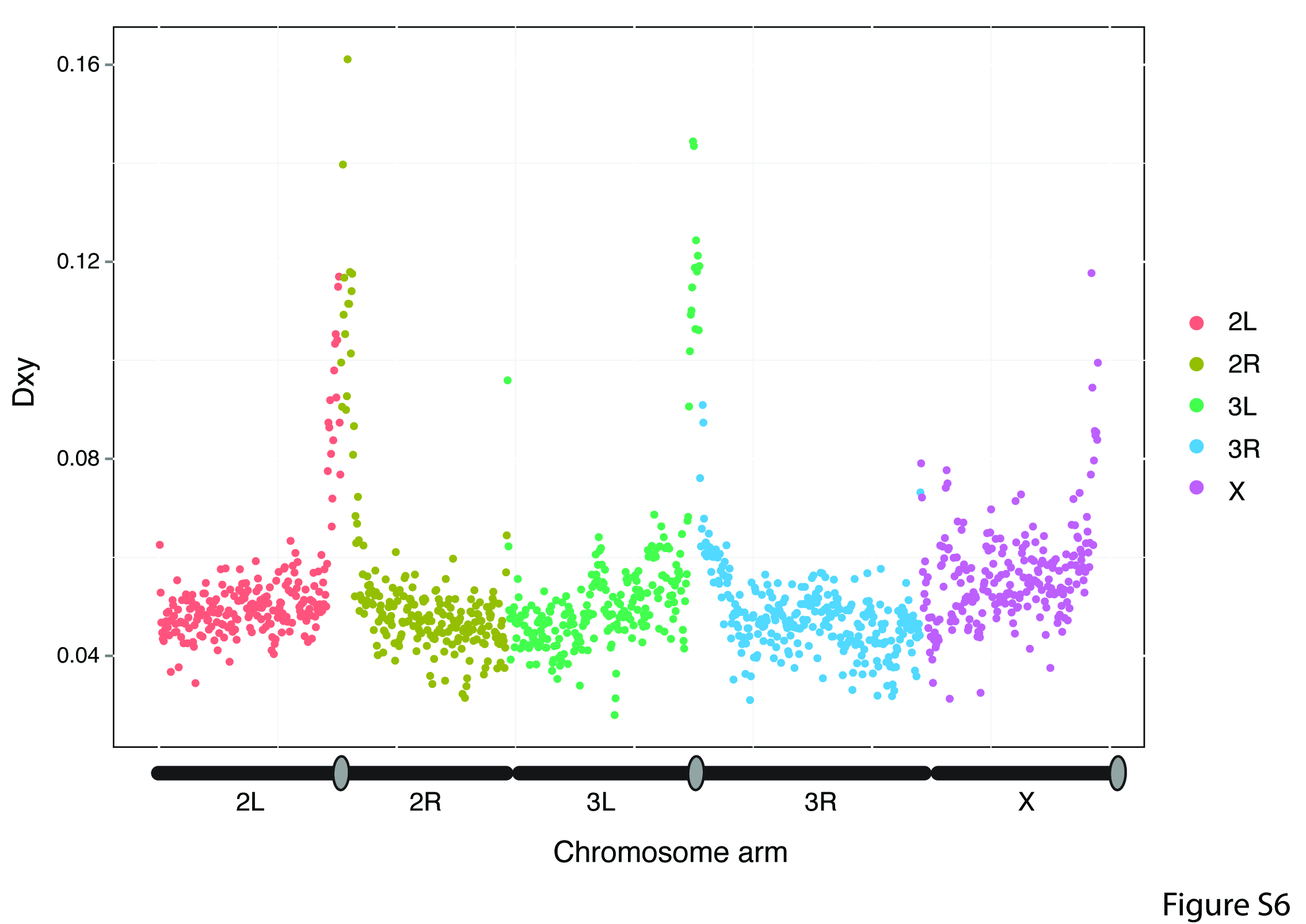

Supplement: Additional file 11: Figure S6. — Genome-wide nucleotide substitution (Dxy) plot for D. simulans and D. melanogaster. Dxy was calculated using 100,000 bp non-overlapping windows. Ovals denote centromeres. (TIF 14250 kb) [file 12862_2015_580_MOESM11_ESM.tif]
